# Laparoscopic vs. open adrenalectomy: perioperative data and survival analysis in 70 dogs with an adrenal tumor

**DOI:** 10.3389/fvets.2023.1156801

**Published:** 2023-08-16

**Authors:** Kirsten L. van Bokhorst, Sara Galac, Hans S. Kooistra, Janny C. de Grauw, Erik Teske, Guy C. M. Grinwis, Sebastiaan A. van Nimwegen

**Affiliations:** ^1^Department of Clinical Sciences, Faculty of Veterinary Medicine, Utrecht University, Utrecht, Netherlands; ^2^IVC Evidensia, Vleuten, Netherlands; ^3^Clinical Sciences and Services, Royal Veterinary College, University of London, London, United Kingdom; ^4^Department of Biomolecular Health Sciences, Faculty of Veterinary Medicine, Utrecht University, Utrecht, Netherlands

**Keywords:** hyperadrenocorticism, hypercortisolism, pheochromocytoma, incidentaloma, recurrence, hypertension, hypotension

## Abstract

Adrenalectomy is the treatment of choice in case of functional adrenal tumors and malignant adrenal incidentalomas. Laparoscopic adrenalectomy (LA) in dogs has gained popularity in recent years, however, clinical studies on large patient populations are scarce. This retrospective study describes perioperative and recurrence data, survival, and prognostic factors in 70 dogs that underwent LA or open adrenalectomy (OA) in our hospital between 2008 and 2022. Diagnosis was based on history, clinical signs, endocrine function tests and advanced diagnostic imaging. Laparoscopic adrenalectomy was performed in 42 dogs (*n* = 27 naturally occurring hypercortisolism, *n* = 4 pheochromocytoma, *n* = 1 pheochromocytoma with concurrent hypercortisolism, *n* = 10 incidentaloma) and OA in 28 dogs (*n* = 22 hypercortisolism, *n* = 3 pheochromocytoma, *n* = 3 incidentaloma). Bilateral adrenalectomy was performed in 8/70 dogs. Surgical duration of LA and OA did not differ significantly in unilateral and bilateral procedures (*P* = 0.108 and *P* = 0.101, respectively). Systemic hypertension occurred in 7/41 and 1/28 dogs during LA and OA, respectively (*P* = 0.130). Hypotension occurred in 2/41 and 4/28 dogs during LA and OA, respectively (*P* = 0.214). A total of 40/42 dogs in the LA group and 27/28 in the OA group survived to discharge (*P* = 0.810). Mean hospital stay was significantly shorter (*P* = 0.006) after LA (1.5 days, range 1–3) than after OA (2.2 days, range 1–4). No significant differences were demonstrated between LA and OA groups in recurrence of adrenal-dependent endocrine disease (*P* = 0.332), disease-free period (*P* = 0.733) and survival time (*P* = 0.353). The disease-specific 1-, 2- and 3-year survival rates were 95, 89, and 89% after LA and 92, 88, and 81% after OA. Tumor size was significantly associated with the occurrence of a recurrence. In addition, tumor size had a negative effect on the disease-free period and survival time. This study shows a favorable outcome of both LA and OA in dogs. Based on low perioperative complication rate, short hospitalization time and long-term outcomes comparable to OA in selected cases, the less invasive laparoscopic approach is considered the preferred technique.

## 1. Introduction

Adrenal tumors (ATs) can either be hormone-secreting, i.e., functional, or hormonally silent. The most common functional adrenocortical tumors (ACTs) in dogs are cortisol-secreting and lead to naturally occurring hypercortisolism (HC), i.e., Cushing's syndrome, whereas less common cortical tumors may secrete aldosterone, sex hormones, or precursor hormones (1). Functional ATs arising from the medulla, i.e., pheochromocytomas (PCCs), lead to catecholamine excess. Furthermore, multiple neoplastic proliferations within one adrenal gland have been reported, and adrenal masses may also represent metastases, granulomas or cysts (2). Hormonally silent ATs are frequently described as ‘incidentalomas' as these tumors are most often diagnosed as an incidental finding during abdominal imaging for other indications (1). Due to greater availability and quality of advanced imaging, these adrenal incidentalomas (AI) are diagnosed with increasing frequency (2, 3).

Adrenalectomy is the treatment of choice when a functional AT causes clinical signs or when an AI is suspected to be malignant, and no metastases have been detected (4–10). Differentiation of benign from malignant ACT in dogs is challenging, but extensive invasion into adjacent tissue or vasculature and tumor size >2 cm are considered consistent with adrenocortical carcinoma (ACC) (2, 11, 12). Hormone excess from functional ATs can predispose to perioperative complications, such as thromboembolism in case of HC and arterial hypertension in case of HC, hyperaldosteronism and PCC. Thorough diagnostic work-up before adrenalectomy is therefore important (2, 9, 11).

Laparoscopic adrenalectomy (LA) has been described in dogs (13–15). In previous reports, LA was successful as treatment of ATs and associated with a low perioperative death rate (10, 16, 17). While laparoscopy is a less invasive technique, concerns exist in human medicine about increased recurrence and reduced disease-free interval after laparoscopic removal of ACC due to incomplete removal or tumor spillage (18, 19). However, in a more recent analysis, no significant differences in recurrence rates or cancer-associated mortality were demonstrated after laparoscopic vs. laparotomic removal of ACC in humans (20). In canine patients, risk factors for the recurrence of endocrine disease after adrenalectomy are not well established. In the study of Taylor et al. (10) no difference in recurrence and long-term survival was demonstrated between 14 LA and 26 OA cases. In that study, intraoperative hypotension occurred less often, and surgical duration was reduced in dogs in which LA was performed. As it is the only study comparing long-term outcome between LA and OA in dogs and performed at one university clinic, more studies are needed to broaden insights on the success rate and recurrence after different adrenalectomy techniques and possible factors related to outcome. Therefore, the current study compares perioperative complications, success- and recurrence rates, long-term survival and related prognostic factors in a large cohort of dogs treated by LA or OA for various ATs.

## 2. Materials and methods

### 2.1. Patient selection

In this retrospective study, the clinical records of dogs that underwent LA or OA at the Department of Clinical Sciences, Faculty of Veterinary Medicine, Utrecht University, the Netherlands, between 2008 and 2022 were reviewed. When laparoscopy was converted to laparotomy, the associated data was analyzed as an OA case.

### 2.2. Diagnosis

Dogs were presented either because of clinical signs of hormone excess, or because of an incidentally discovered adrenal mass at diagnostic imaging for reasons not specifically related to endocrine disease. All dogs included in the study underwent a thorough diagnostic evaluation. Preoperative diagnosis was based on the history, clinical signs, hematology, biochemistry and urinalysis, combined with hormone analysis to evaluate AT functionality. In dogs with a history, physical examination and laboratory findings consistent with HC, the diagnosis was confirmed by means of endocrine function tests, i.e. low-dose dexamethasone suppression test (LDDST) or urinary corticoid-to-creatinine ratios (UCCRs) combined with oral high-dose dexamethasone suppression test (HDDST), and endogenous plasma ACTH measurement. Endocrine function tests and hormone analyses for HC were performed and interpreted as described previously (21, 22). Plasma aldosterone concentration and plasma or urine metanephrines were measured to test for hyperaldosteronism and PCC, respectively, as described previously (23, 24).

### 2.3. Adrenal imaging

Diagnostic imaging of the pituitary, thorax and abdomen was performed in all dogs included in this study. Computed tomography (CT) imaging was performed under general anesthesia with a single or multiple slice helical CT scanner (Secura CT Scanner; Phillips, Best, the Netherlands), using a protocol described previously (25). The structure, shape and symmetry of both adrenals and tumor invasion into blood vessels was evaluated by a board-certified radiologist. Adrenal tumors were measured in three dimensions, i.e. length, height and width in centimeters, using Agfa Impax computer software (Impax Agility 8.1.2; Agfa-Gevaert, Mortsel, Belgium) or Radiant DICOM viewer (Medixant; version 2021.2, Poznan, Poland) tools. Maximum AT dimension was determined, and AT volume was estimated by using the formula for calculation of the volume of an ellipsoid: 4/3 × Pi × A × B × C, where A, B and C are the lengths of all three semi-axes of the ellipsoid, i.e. 0.5 times the measured diameters of the adrenal gland in each dimension. CT images were evaluated for the presence of masses consistent with metastases. Depending on the specific location of the mass(es), ultrasound-guided fine needle aspiration biopsies were taken for cytologic evaluation preoperatively, or tissue biopsies were taken during surgery for histologic evaluation.

Based on integration of clinical, laboratory and diagnostic imaging findings, a preoperative endocrine diagnosis of HC, hyperaldosteronism, PCC or combinations thereof was made. If clinical signs and results of endocrine function tests were not consistent with one of these diagnoses, the AT was defined as AI. Preoperative medical treatment for HC or PCC was recorded.

### 2.4. Adrenalectomy

Anesthesia was supervised by a board-certified anesthesiologist. The anesthetic protocol was not standardized, however generally consisted of IV or IM pre-medication with a mu-opioid and an antimuscarinic agent, IV induction with propofol, and maintenance with isoflurane in oxygen (FiO_2_ ~50%) titrated to maintain adequate anesthetic depth, with constant rate infusion of fentanyl (Fentadon, Dechra, Bladel, the Netherlands) 5–20 mcg/kg/h or sufentanil (Sufentanil-hameln, Hameln pharma GMBH, Hameln, Germany) 0.5–2 mcg/kg/h at the anesthetist's discretion. Decisions to adjust the anesthetic plan were based on the attending clinician's judgement of the clinical parameters of the dog. Parameters of respiratory and cardiovascular function were documented every 5–10 min, as were any comments on anesthesia or surgery. Dogs diagnosed with HC or AI received intravenous hydrocortisone (Solu-Cortef, Pfizer, Capelle a/d IJssel, the Netherlands) 1 mg/kg over 6 h after induction. If bilateral adrenalectomy was performed, desoxycortone acetate (Pharmacy Faculty of Veterinary Medicine, Utrecht University, the Netherlands) 0.10 mg/kg q24h SC was added to the supplementation protocol. Surgical duration was calculated from first incision to completion of abdominal closure.

During anesthesia, blood pressure was measured invasively via an arterial catheter connected to an electronic pressure transducer, or, if catheter placement was unsuccessful, via non-invasive oscillometric measurement (Pettrust, Taoyuan County, Taiwan). Systemic hypertension was defined as a systolic arterial pressure (SAP) >160 mm Hg sustained for a minimum of 10 min (10, 26). In addition, SAP was scored as borderline elevated when blood pressure was above 140 mm Hg for a minimum of 10 min but did not persistently exceed 160 mm Hg. Hypotension was defined as a mean arterial blood pressure (MAP) <60 mm Hg sustained for a minimum of 10 min. Systemic hyper- or hypotension was treated as deemed necessary by the attending anesthetist, by means of adjustment of inhalant agent concentration, provision of additional analgesia, IV fluid boluses and/or provision of vasoactive drugs [alpha- or beta-adrenergic antagonists (esmolol, phentolamine) or -agonists (dobutamine, phenylephrine, norepinephrine)].

Adrenalectomy was performed by the same surgeon (SN) for each dog based on methods described previously (9, 13, 27). In principle, there was no tumor size limit when considering LA or OA as long as the tumor shape was relatively clearly marginated without overt involvement of important blood vessels precluding complete tumor excision. Hence, irregular tumors with extensive irregular invasion in or around surrounding tissues or encasing aorta, vena cava, or cranial mesenteric artery were excluded. In addition, tumor thrombus extension in the vena cava beyond hepatic hilus was considered an exclusion criterium. The decision to perform LA was based on imaging findings, including a relatively well-defined and smoothly marginated AT, no visible vascular invasion beyond the phrenicoabdominal vein (i.e., no tumor thrombus entering vena cava or renal vein), no tumor infiltration in the kidney or renal hilus, and subjective assessment of AT size in relation to expected surgical space which is influenced by the size and conformation of the dog. If considered possible, LA was the preferred technique.

**Figure 1:**
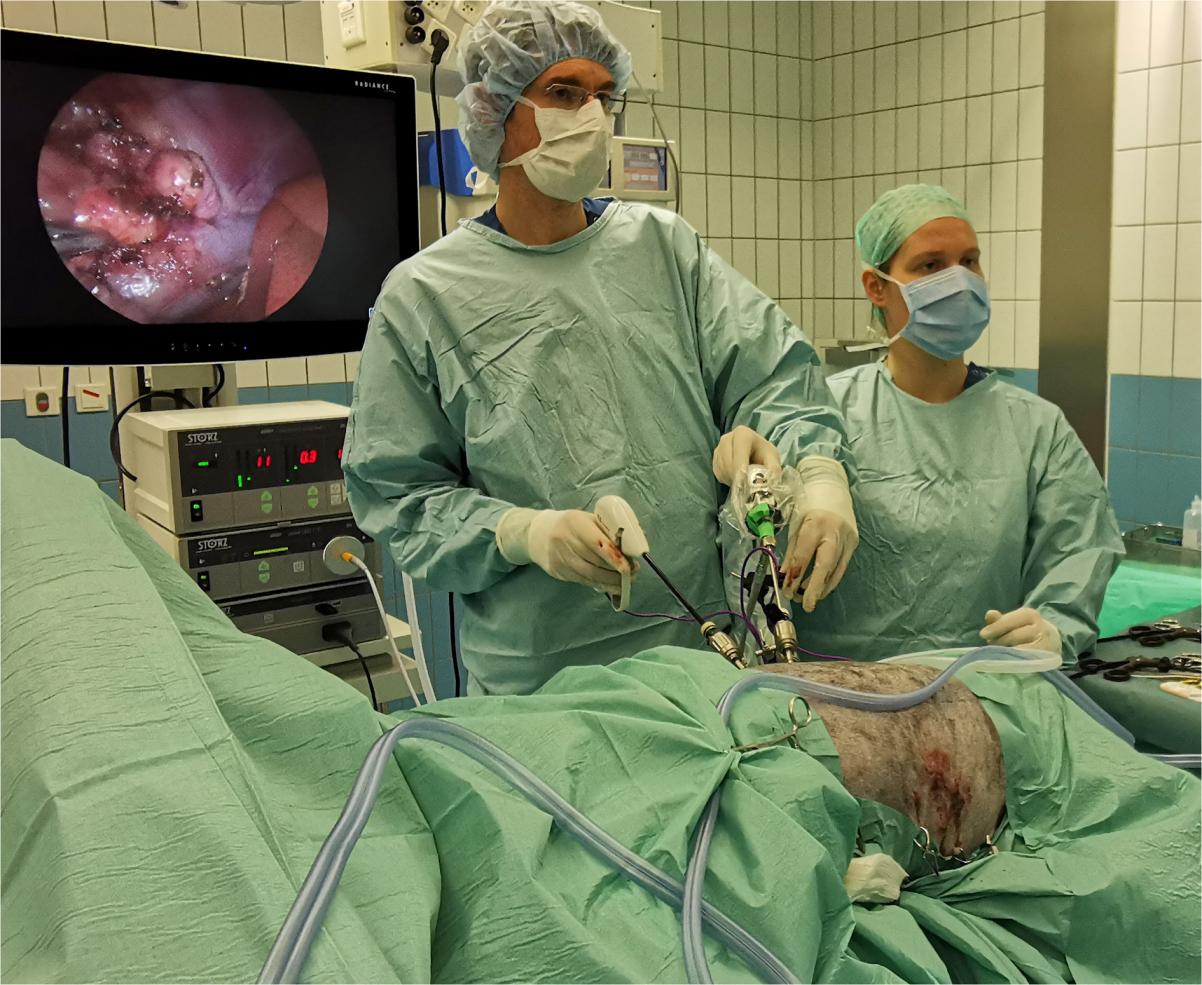
Operation room setting during bilateral laparoscopic adrenalectomy through flank approach in sternal recumbency in an Australian Shepherd dog with HC. In the picture, right LA is in progress and left LA has been completed (with 3 portals sutured). Both procedures are performed consecutively without repositioning or re-draping the patient.

For LA the lateral body wall was widely clipped and aseptically prepared from midway the thorax to tuber coxae in craniocaudal orientation and from dorsal midline to ventral midline in dorsoventral orientation allowing conversion to a paracostal laparotomy approach should this become necessary. Dogs were placed in sternal recumbency, up to 30^o^ contralateral oblique, tightly supported by moldable vacuum cushions positioned under the sternum and between the hind legs in order to lift the sternum and pubic bone to allow the abdomen to hang in between, promoting abdominal organs to move dependently (28). A motorized table allowed further tilting of the patient in any direction if needed during adrenalectomy. In most cases, a modified Hasson technique using 6 mm diameter threaded cannula (Ternamian Endotip, Karl Storz, Tuttlingen, Germany) was used for insertion of the 1st camera port 3–5 cm caudal to the last rib and 2–3 cm ventral to the transverse spinous processes (lateral to the kidney). In a few early cases a Veress needle was inserted just caudal to the 13th rib in the paralumbar fossa. After abdominal insufflation with CO_2_ (intra-abdominal pressure of 8 mmHg), two additional 6-mm cannulas were placed cranial and caudal to the camera portal, based on intra-abdominal findings, aiming for triangulation of instruments. A 5-mm diameter 30° laparoscope (Karl Storz, Tuttlingen, Germany) was used and tissue manipulation and dissection was performed using either a blunt tip grasping-, Babcock-, or Kelly-type 5-mm diameter laparoscopic forceps (Clickline^(R)^, Karl Storz, Tuttlingen, Germany). LigaSure^TM^ Maryland tissue sealer/divider with ForceTriad^TM^ generator (Medtronic, Eindhoven, the Netherlands) was used for tissue dissection, sealing and transection in the majority of cases; LigaSure-V was used in a few early cases. To achieve access to the right adrenal gland, the right hepatorenal ligament was incised and the lateral hepatic lobe was retracted cranially. To gain proper exposure of the left and right adrenal gland, the kidney was retracted caudally and the peritoneum between adrenal gland and kidney was dissected close to the adrenal capsule, taking care not to damage the phrenicoabdominal vein. If needed, exposure of the adrenal gland could be further improved by increased retraction of the kidney after dissecting the craniodorsal peritoneal attachments of the kidney. Further dissection of the adrenal gland from its peritoneal attachments was performed with minimal manipulation of the AT from caudodorsal to craniodorsal, dissecting and transecting the dorsal branch (and with left-sided AT also the ventral branch) of the phrenicoabdominal vein with LigaSure. If a (small) tumor thrombus was present in the phrenicoabdominal vein, care was taken to include the complete thrombus with the portion of the vein that was excised en-bloc with the AT. Circumferential dissection of the adrenal gland was continued with careful dissection from its fibrous and nervous attachments and blood vessels medially and caudally, taking care not to damage the renal vein that may by situated close to, or adhered to, the tumor. On the right side, the ventral part of the adrenal gland was carefully dissected from the wall of the vena cava while gently lifting or pushing the gland dorsally. The ventral branch of the right phrenicoabdominal vein was dissected free, and, while gently lifting the AT dorsally, the phrenicoabdominal vein was sealed and transected at the level of the vena cava, ensuring that, if present, the small tumor thrombus was contained in the excised phrenicoabdominal vein. Capsular penetration was recorded if this occurred despite meticulous dissection. The excised adrenal gland was removed from the abdomen through an enlarged portal using a retrieval bag or glove-finger to prevent possible contamination with tumor cells. In bilateral LA, patients were clipped and aseptically prepped circumferential and positioned in sternal recumbency as outlined before, ensuring accessibility of both lateral flanks without repositioning the patient between procedures (Figure 1). LA was started on the left side and the procedure was repeated for the contralateral side as described previously. The abdomen was decompressed after inspection for hemorrhage, and the laparoscopic port sites were closed routinely in three layers.

Open adrenalectomy was performed via paracostal or ventral midline celiotomy, depending on individual tumor-related anatomy and vascular involvement. Tumors without apparent involvement of organs or blood vessels medial to the tumor or right-sided tumors with caval invasion limited to the abdominal vena cava were usually approached via paracostal flank celiotomy. Larger tumors growing into the renal hilus, with suspicion of renal vein involvement, left-sided tumors with suspicion of caval invasion and bilateral adrenalectomies were approached through ventral midline celiotomy. Dogs were positioned in dorsal or lateral recumbency depending on the elected celiotomy approach. After standard celiotomy and exposure of the adrenal gland, removal was initiated by dissection of all peritumoral attachments with a combination of sharp, blunt, and electrosurgical dissection using a combination of standard monopolar and bipolar electrosurgery and LigaSure^TM^ small jaw open sealer/divider. The phrenicoabdominal vein, including tumor thrombus if present, was isolated, sealed and divided at the lateral aspect of the gland using LigaSure^TM^, suture, or vascular clips. The adrenal gland was further dissected of surrounding fascial attachments and other structures, including adhered blood vessels and vena cava if indicated. Before excision, the phrenicoabdominal vein was ligated or sealed and divided near its entry into the vena cava. Caval tumor thrombi were removed en-bloc with the AT through a caval venotomy at the level of the phrenicoabdominal vein. Rummel tourniquets were always placed on vena cava pre- and post-thrombus and on any renal vein connected to the affected caval part. If tumor thrombus size permitted, a Satinsky vascular clamp was pre-placed before venotomy and Rummel tourniquets were not tightened. For larger tumor thrombi, temporary complete caval flow occlusion was performed by tightening the Rummel tourniquets for cavotomy and tumor thrombus removal. After thrombus removal, a Satinsky vascular clamp was always placed over the cavotomy site and cavotomy suturing with 5–0 or 6–0 polypropylene was performed under partial occlusion of cava. After adrenalectomy the celiotomy incision was closed routinely in three layers.

Postoperatively, dogs were carefully monitored and treated for signs of discomfort and any hemodynamic or electrolyte derangements in the Intensive Care Unit (ICU). Opioid analgesia was continued and tapered at discretion of the attending intensivist, based on clinical examination findings including pain assessment. In dogs diagnosed with HC or AI, glucocorticoid supplementation was continued by means of oral cortisone acetate (Teva, Harlem, the Netherlands) 1 mg/kg q12h PO once the dog started to eat. Cortisone acetate was tapered in 7–8 weeks and discontinued after unilateral procedures, whereas after bilateral adrenalectomy, glucocorticoid and mineralocorticoid (fludrocortisone; Fendigo, Brussels, Belgium/Florinef, Aspen, the Netherlands) 6.25 mcg/kg q12h PO supplementation was continued for life as is done in dogs with primary hypoadrenocorticism. Dogs were discharged from the hospital when they were considered comfortable on oral analgesics and demonstrated sufficient voluntary food intake.

### 2.5. Histopathology

Histopathological examination of hematoxylin and eosin-stained specimens was performed by a board-certified pathologist to identify and differentiate adrenocortical from medullary neoplasia. The neoplasms were evaluated using a set of up to twenty morphological characteristics including cell and nuclear morphology, necrosis, vascular invasion and mitotic index (29, 30). If deemed possible by the attending pathologist, a morphological conclusion of adrenocortical adenoma (ACA) vs. ACC was drawn. The presence of a PCC was confirmed by performing immunohistochemistry for chromogranin A and synaptophysin (31).

### 2.6. Follow-up

Follow-up information was collected from the medical files and by contacting owners and referring veterinarians. Recurrence was defined as reappearance of clinical signs of the original adrenal disease and positive endocrine function tests, and, when clients consented, imaging as described for the initial work-up.

### 2.7. Statistics

Statistical analysis was performed using commercial statistical software (SPSS 27.0.1.0 for Windows, SPSS). The Kolmogorov-Smirnov test was used to assess normality of the data and the Levene's test for equality of variances. Perioperative variables compared between LA and OA included age, body weight, AT size, vascular invasion, surgical duration, occurrence of hyper- or hypotension, intraoperative capsular penetration, survival to discharge and hospitalization time. Normally distributed data was compared using *T*-tests. If data was not normally distributed or variances not equal, Mann–Whitney *U*-test was used to compare groups. Continuous variable comparison between multiple groups was performed by one-way analysis of variance followed by *post-hoc* tests. Nominal variables were compared between groups using cross-tabulation and Chi-square tests, or Fisher's exact tests if the number of animals in a particular group was smaller than 10.

Survival curves were drawn by the Kaplan–Meier method. Univariate tests for comparison of LA and OA survival data were made using log-rank testing and proportional hazard logistic regression models. Univariate variables with *P* < 0.20 were introduced in a multivariate Cox proportional hazards model regression analysis of disease-free period (DFP) and survival time with forward stepwise selection. Disease-free period was calculated from time of surgery to occurrence of recurrence. For the DFP analysis, dogs were censored if they were lost to follow-up, they died before recurrence, or recurrence had not occurred before the end of the study period. Survival times were calculated from the time of surgery to tumor-associated death. If a dog died from an unrelated cause, was lost to follow-up, or was still alive at the end of the study it was censored and the last known date that the dog was still alive was used as censoring date. Binomial logistic regression analysis was performed to ascertain the effects of age, body weight, type of surgery, vascular invasion, lesion side, maximal AT dimension, AT volume, surgery duration, intraoperative capsular penetration, histopathologic diagnosis, systemic hypertension and hypotension on recurrence.

## 3. Results

### 3.1. Animals and diagnosis

Laparoscopic adrenalectomy was performed in 45 dogs; in 3 dogs LA was converted to laparotomy, resulting in a total of 42 dogs in the LA group (*n* = 19 left-sided, *n* = 18 right-sided, *n* = 5 bilateral). In 25 dogs a primary laparotomy approach was used, resulting in a total of 28 dogs in the OA group (*n* = 13 left-sided, *n* = 12 right-sided, *n* = 3 bilateral). The proportion of left-sided, right-sided and bilateral adrenalectomies was not significantly different between LA and OA (*P* = 0.987).

Median body weight of dogs in the LA group (15.2 kg, range 6.5–50.3 kg) did not differ significantly from the dogs in which OA was performed (14.0 kg, range 5.1–43.6 kg; *P* = 0.278). Most dogs in the LA group were neutered (*n* = 32; 16 males, 16 females), 10 were intact (8 males, 2 females) and the most common breeds were mixed breed (*n* = 9), Labrador retriever (*n* = 5) and Shih tzu (*n* = 3). The OA group consisted of 23 neutered dogs (9 males, 14 females) and 5 intact dogs (4 males, 1 female) and the most common breeds were mixed breed (*n* = 7) and Shih tzu (*n* = 3). Mean age at LA was 9.9 years (SD 1.8) and did not differ significantly from OA cases [9.6 years (SD 1.2); *P* = 0.415].

Preoperative endocrine diagnoses in the LA group were HC (*n* = 27), PCC (*n* = 4), PCC with concurrent HC (*n* = 1) and AI (*n* = 10). In the OA group, preoperative endocrine diagnoses were HC (*n* = 22), PCC (*n* = 3) and AI (*n* = 3). Incidentalomas were discovered during ultrasonography performed for various reasons including vomiting, diarrhea, or mild liver enzyme elevation at preanesthetic screening. When specifically asked, owners of 9/13 dogs with AI reported increased water intake. In at least 3/9 dogs, this was diagnosed as primary polydipsia based on urinary specific gravity ≥1.035 without glucosuria. In one dog diagnosed with AI no plasma metanephrines were measured due to technical limitations. In two dogs with AI, plasma ACTH concentrations were suppressed (<5 pg/mL) and the contralateral adrenal gland small in size. In one of these dogs the adrenal mass was an incidental finding during diagnostic work-up for diarrhea, while the other dog presented with polyuria, polydipsia and intermittent polyphagia. In the latter dog, LDDST revealed low plasma cortisol concentrations (24, 3 and 2 nmol/L before and 4- and 8 h after intravenous dexamethasone, respectively); this dog therefore did not meet the criteria of HC and was thus diagnosed with AI. Reasons for bilateral adrenalectomy were HC with bilateral adrenal neoplasia in 6 dogs and unilateral AT with concurrent pituitary-dependent HC in 2 dogs. In the two dogs with concurrent pituitary-dependent HC, hypophysectomy was not deemed a preferrable option in addition to unilateral adrenalectomy, as these comprised small-sized pituitary tumors in dogs of 11 and 12 years of age.

Six out of 28 and 3/22 dogs with HC were treated with trilostane (Vetoryl, Dechra, Bladel, the Netherlands) 0.5–1.5 mg/kg q12h PO before LA and OA, respectively. Trilostane treatment was discontinued for a minimum of 3 days before procedures under anesthesia. All dogs with confirmed PCC, including the Cocker spaniel with concurrent HC, were treated with phenoxybenzamine (Dibenzyran, Aristo pharma GmbH, Berlin) 0.25–0.5 mg/kg q12h PO in the 2 weeks before surgery. In addition, one Shih Tzu dog diagnosed with AI but high-normal plasma normetanephrine concentration (3.24 nmol/L, reference range 0.90–3.56) (24) was treated with phenoxybenzamine preoperatively.

### 3.2. Adrenal imaging

Based on preoperative imaging, median maximal AT dimension (2.7 cm, range 1.5–5.2) and AT volume (3.06 cm^3^, range 0.77–41.4) in the LA group were smaller than in the OA group [3.2 cm (range 1.7–9.9) and 6.03 cm^3^ (range 1.6–374.3); *P* = 0.037 and *P* < 0.001, respectively]. Results of preoperative CT scans were suspicious of either caudal vena cava or phrenicoabdominal vein invasion in 11 dogs with HC, 2 dogs with PCC and 1 dog with AI. Vascular invasion was not significantly associated with maximum AT dimension (*P* = 0.639) or AT volume (*P* = 0.097). In 2/14 dogs, both with phrenicoabdominal vein invasion, LA was performed, whereas OA was performed in the remaining 12/14 dogs.

### 3.3. Adrenalectomy

Mean surgical duration of LA was 138 min (SD 52) in unilateral and 230 min (SD 24) in bilateral procedures, which was not significantly different from OA [unilateral 153 min (SD 36), bilateral 197 min (SD 22); *P* = 0.108 and *P* = 0.101, respectively]. Surgical time did not differ significantly between LA and OA for left-sided (*P* = 0.891), right-sided (*P* = 0.149) and bilateral procedures (*P* = 0.102). However, for both techniques combined, left-sided adrenalectomy duration (mean 132 min, SD 42) was shorter than right-sided (157 min, SD 48; *P* = 0.033).

Systemic blood pressure evaluation during anesthesia was available in 69/70 dogs; 41/42 dogs that underwent LA and all 28 dogs in which OA was performed. In the remaining miniature dachshund with HC, arterial cannulation was unsuccessful and oscillometric monitoring failed. Blood pressure was measured invasively in 49/69 dogs (*n* = 28 LA and *n* = 21 OA) and non-invasively in 20/69 dogs (*n* = 13 LA and *n* = 7 OA). Systemic blood pressure categories per endocrine diagnosis and surgical technique are summarized in Table 1. Systemic hypertension occurred in 7/41 and 1/28 dogs during LA and OA, respectively (*P* = 0.130). Borderline elevated systemic blood pressure was noted in 9/41 and 6/28 dogs during LA and OA, respectively (*P* = 0.999). Hypotension occurred in 2/41 and 4/28 dogs during LA and OA, respectively (*P* = 0.214). Alpha- or beta-adrenergic agonists were used in two other dogs which did not develop systemic hypotension. One dog diagnosed with PCC (OA) and one dog with a left-side PCC and concurrent pituitary dependent HC (bilateral LA), initially developed hypertension and short bouts of SAP >160 mm Hg, respectively, until removal of the AT; thereafter hypotension occurred which required a vasopressor to increase MAP > 60 mm Hg.

**Table 1:** Systemic blood pressure categories per endocrine diagnosis in 41 dogs that underwent laparoscopic adrenalectomy and 28 dogs that underwent open adrenalectomy.

**Blood pressure**	**Endocrine diagnosis**	**LA (*n =* 41)**	**OA (*n =* 28)**	**Total**
Systemic hypertension (SAP >160 mm Hg)	HC	*N =* 5	*N =* 1	6/48
	PCC	*N =* 1	*N =* 0	1/7
	PCC + HC	*N =* 0	*N =* 0	0/1
	AI	*N =* 1	*N =* 0	1/13
Borderline elevated blood pressure (SAP 141–160 mm Hg)	HC	*N =* 6	*N =* 4	10/48
	PCC	*N =* 0	*N =* 2	2/7
	PCC + HC	*N =* 0	*N =* 0	0/1
	AI	*N =* 3	*N =* 0	3/13
Systemic hypotension (MAP <60 mm Hg)	HC	*N =* 1	*N =* 2	3/48
	PCC	*N =* 0	*N =* 2	2/7
	PCC + HC	*N =* 1	*N =* 0	1/1
	AI	*N =* 0	*N =* 0	0/13

LA, laparoscopic adrenalectomy; OA, open adrenalectomy; SAP, systolic arterial pressure; MAP, mean arterial pressure; HC, hypercortisolism; PCC, pheochromocytoma; AI, adrenal incidentaloma.

Caval tumor thrombus extension in OA were all pre-hepatic or just entering hepatic hilus, with largest thrombi not extending cranially beyond the first hepatic lobar vein branches. Intraoperative capsular penetration was reported in 19 dogs (*n* = 13 LA, *n* = 6 OA). No association was found between capsular penetration and surgical approach (*P* = 0.418), maximal AT dimension (*P* = 0.516) or AT volume (*P* = 0.342).

Adrenalectomy was successfully performed without major intraoperative complications in all but one dog. In this Jack Russell terrier, no systemic hypertension was present preoperatively and endocrine function tests excluded HC and hyperaldosteronism. It was this dog in which no plasma metanephrines could be determined preoperatively due to technical limitations and the dog was not pretreated with phenoxybenzamine. The dog underwent LA for a right-sided suspected AI measuring 1.2 × 1.1 × 2.1 cm and died after developing uncontrollable systemic hypertension (SAP >300 mm Hg) during surgery leading to cardiac arrest. During anesthesia for the CT scan 7 weeks before surgery, no anesthetic complications occurred. Postmortem histopathology was declined by the owner and therefore it was not possible to make a definitive diagnosis in this dog.

In two dogs in which unilateral LA was initiated, surgery was converted to OA. Reasons for conversion were moderate hemorrhage due to tumor capsule laceration medial to the tumor in one dog and diffuse hemorrhage from the renal capsule in another small (5.1 kg) dog with limited surgical space, which could not be controlled laparoscopically. In one dog that underwent adrenalectomy for bilateral adrenal-dependent HC, surgery had to be converted to an open technique for the second (right) adrenal because of inadvertent pneumothorax during canula placement, leading to obscured visibility by the caudally displaced diaphragm. In addition, accidental pneumothorax occurred in a 7.9 kg dog due to inadvertent puncture of the thoracic cavity during Veress needle placement. In this case, the pneumothorax was managed intraoperatively with thoracic drainage and the LA procedure was completed without further events.

### 3.4. Histopathology

Adrenal histopathology was available in all 69/70 dogs in which adrenalectomy was successful. Pheochromocytoma was confirmed in all 8 dogs with this preoperative diagnosis. In the remaining 61 dogs, cortical tumors were diagnosed, as summarized in Table 2.

**Table 2:** Histopathologic diagnoses of cortical neoplasia in 36 dogs that underwent laparoscopic adrenalectomy and 25 dogs that underwent open adrenalectomy.

**Preoperative endocrine diagnosis**	**Histologic diagnosis**	**LA (*n =* 36)**	**OA (*n =* 25)**	**Total**
Adrenal-dependent HC - unilateral adrenalectomy	ACC	*N =* 17	*N =* 14	31
	ACA	*N =* 4	*N =* 3	7
	Undefined	*N =* 1	*N =* 2	3
Adrenal-dependent HC - bilateral adrenalectomy	ACC	*N =* 0	*N =* 1	1
	ACC + ACA	*N =* 1	*N =* 0	1
	ACA	*N =* 2	*N =* 2	4
Adrenal tumor with concurrent pituitary-dependent HC	ACCs (bilateral adrenalectomy)	*N =* 1	*N =* 0	1
	ACA (unilateral adrenalectomy)	*N =* 1	*N =* 0	1
AI	ACC	*N =* 3	*N =* 0	3
	ACA	*N =* 4	*N =* 3	7
	Undefined	*N =* 2	*N =* 0	2

LA, laparoscopic adrenalectomy; OA, open adrenalectomy; HC, hypercortisolism; AI, adrenal incidentaloma; ACC, adrenocortical carcinoma; ACA, adrenocortical adenoma.

In one dog with confirmed PCC and negative endocrine function tests for HC, a concurrent cortical tumor was found in the same adrenal gland. In another dog with confirmed adrenal-dependent HC and plasma metanephrines within the reference range, a concurrent medullary tumor consistent with PCC was demonstrated within the same adrenal gland.

Of the 14 dogs in which preoperative CT images were suggestive of vascular invasion, adrenal histopathology was consistent with PCC in 2 dogs, ACC in 9 dogs, and ACA in 3 dogs including the dog with a preoperative endocrine diagnosis of AI. In dogs with ACT, vascular invasion was not more common in histologic carcinomas than in adenomas (*P* = 0.732).

### 3.5. Follow-up

A total of 40/42 dogs in the LA group and 27/28 in the OA group survived until discharge (*P* = 0.810). In addition to the dog that died during surgery, two dogs died shortly after recovery. One dog developed vomiting, diarrhea and dyspnea after recovery from LA on the ICU and went into cardiac arrest within 12 h post-operatively. This dog underwent surgery for HC due to a left-sided AT and no immediate cause of death, including pulmonary thromboembolism, was found at necropsy. Another dog underwent OA for bilateral adrenal-dependent HC recovered uneventfully initially but went into cardiac arrest thereafter, while thrombocyte count and clotting times were within their respective reference ranges and no free fluid was found at abdominal ultrasound. Resuscitation was unsuccessful and postmortem examination was declined in this dog.

In dogs surviving to discharge, mean hospital stay after adrenalectomy was significantly shorter in the LA (1.5 days, range 1–3) compared to the OA group (2.2 days, range 1–4; *P* = 0.006). One dog with adrenal-dependent HC and diabetes mellitus was excluded from this analysis as it developed a catheter-site infection and was hospitalized for an extended period of 11 days unrelated to LA.

**Figure 2:**
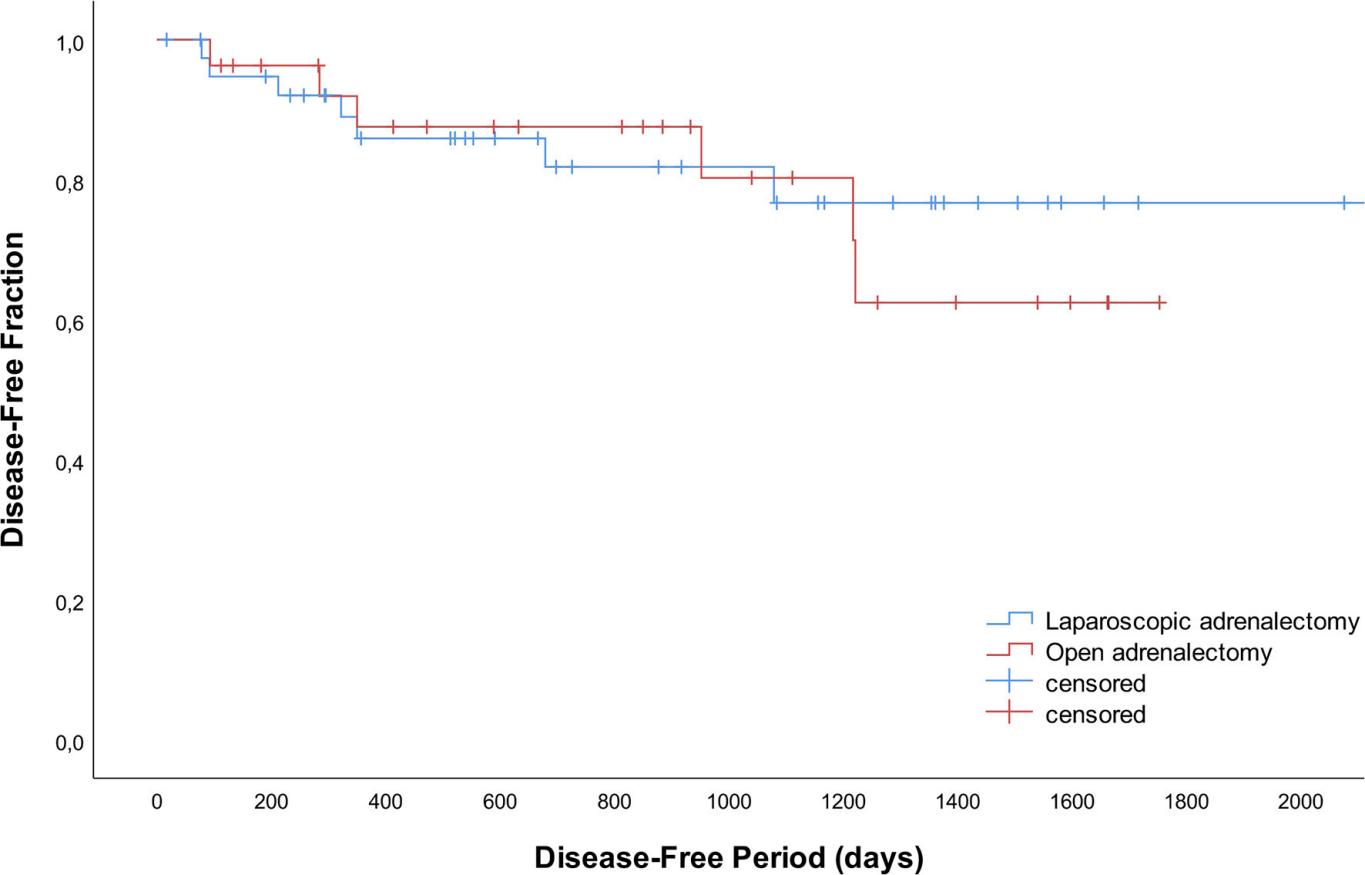
Disease-free curves for 42 dogs that underwent laparoscopic adrenalectomy and 28 dogs that underwent open adrenalectomy.

Recurrence occurred in 13 dogs with adrenal-dependent HC; 7 after LA (*n* = 3 right, *n* = 3 left, *n* = 1 bilateral) and 6 after OA (*n* = 1 right, *n* = 5 left; *P* = 0.332) after a mean of 533 days (SD 438). In addition to clinical signs consistent with HC and positive endocrine function testing, diagnostic imaging was performed in 4/13 dogs (*n* = 3 thoracic radiographs and abdominal ultrasound, *n* = 1 CT scan of the skull, thorax and abdomen) confirming either distant metastases (*n* = 3) or local abnormalities suggestive of tumor regrowth (*n* = 1). In two dogs (*n* = 1 LA, *n* = 1 OA) the liver was biopsied during surgery for histopathologic evaluation of small nodules visible during laparoscopy and laparotomy, respectively. Adenocarcinoma metastases were confirmed, and clinical signs of HC reoccurred after 3 months. Disease-free periods did not differ significantly between LA and OA (Figure 2; *P* = 0.733).

**Figure 3:**
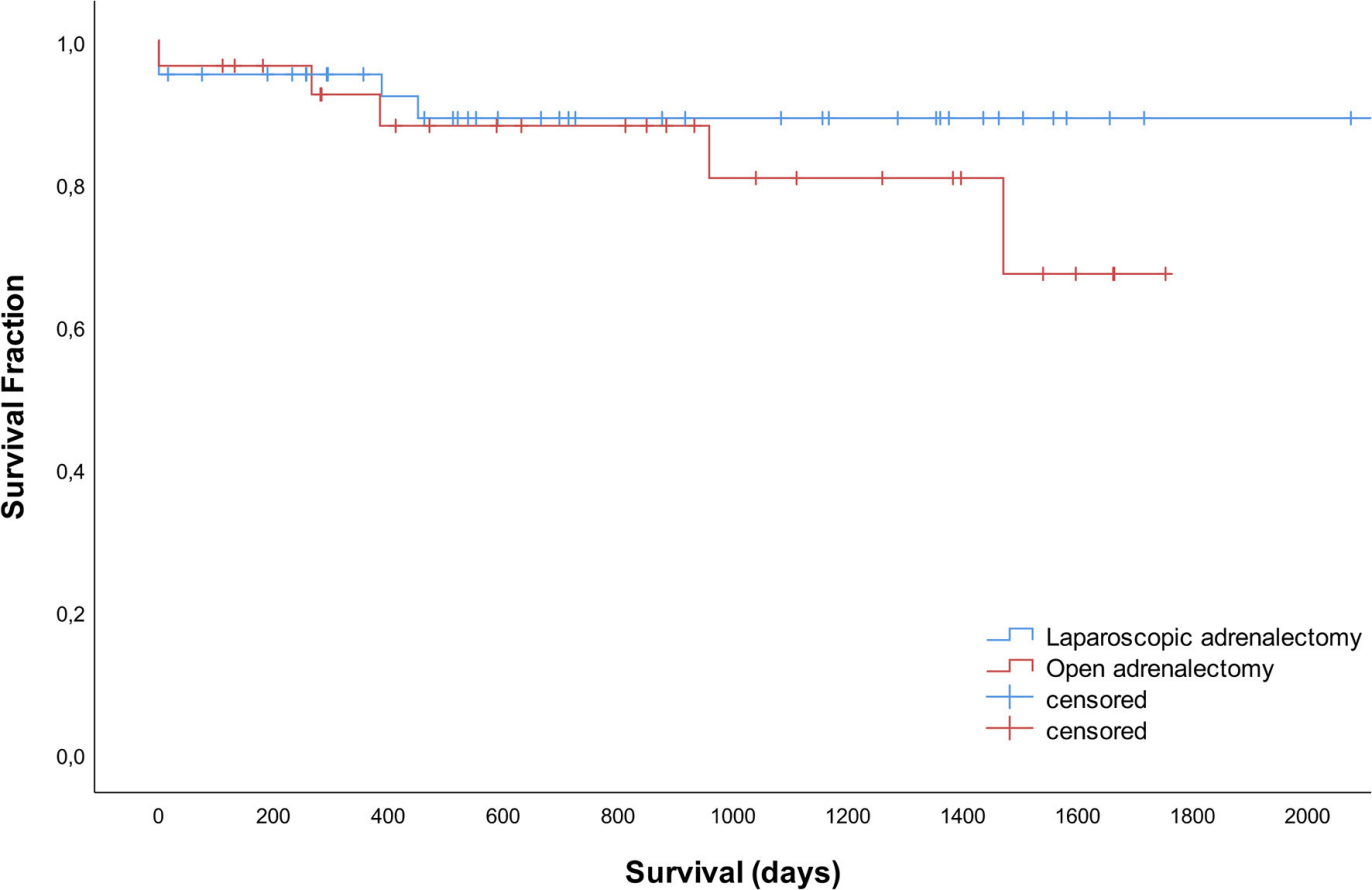
Survival curves for 42 dogs that underwent laparoscopic adrenalectomy and 28 dogs that underwent open adrenalectomy.

Of the total group, 42 dogs died during the study period, 5 dogs were lost to follow up and 23 were still alive at the time of manuscript preparation. Survival times did not differ significantly between LA and OA groups (*P* = 0.353). Median survival times were not reached. The disease-specific 1-, 2- and 3- year survival rates were 95, 89, and 89% after LA and 92, 88, and 81% after OA (Figure 3).

Based on univariate regression analysis the parameters body weight, maximal AT dimension, AT volume and surgery duration were introduced in a multivariate Cox proportional hazard regression model to predict DFP and overall survival time. Maximal AT dimension appeared to be the only significant negative predictor (*P* = 0.001) for DFP and AT volume (*P* = 0.003) for overall survival.

Vascular invasion was relatively rare in the 13 dogs in which recurrence was diagnosed, with invasion of the caudal vena cava visible on the preoperative CT scan in two dogs and invasion of the phrenicoabdominal vein in one dog. Vascular invasion (*P* = 0.829) and perioperative capsular penetration (*P* = 0.725) were not more common in dogs with recurrence. Recurrence was associated with larger AT volumes both in LA (*P* = 0.008) and OA (*P* = 0.021), and for all adrenalectomies considered together (*P* = 0.001). In all 13 dogs with recurrence, histopathology of the adrenal gland(s) was consistent with ACC.

In the binomial logistic regression analysis to predict recurrences, a model including the parameters maximum tumor dimension and histopathologic diagnosis was constructed. The logistic regression model was statistically significant, χ^2^(2) = 26.804; *P* < 0.001. The model explained 55.6% (Nagelkerke R^2^) of the variance in recurrences and correctly classified 85.0% of cases. Increasing maximum tumor dimension was associated with an increased likelihood of recurrence, with odds of recurrence 3.9 higher for each cm increase in maximum tumor dimension size compared to the smallest one.

Two dogs were lost to follow-up after diagnosis of recurrence, 2 were still alive and 9 died. Of the dogs that died, death was related to recurrence of adrenal-dependent HC in 6/9 dogs. One of the dogs with recurrence showed suppression of the UCCR after oral dexamethasone at initial diagnosis, suggestive of pituitary-dependent HC. However, endogenous ACTH was suppressed and diagnostic imaging findings consistent with ATs. In this specific dog, metastatic ACC cells were demonstrated in fine-needle aspiration biopsies of the liver 2 years after bilateral LA. In addition to recurrence defined by reappearance of the initial adrenal-dependent endocrinopathy, food-dependent HC was diagnosed in one dog which developed clinical signs of HC after initial improvement after unilateral adrenalectomy. Another dog with adrenal-dependent HC and suppressed endogenous ACTH at diagnosis was diagnosed with a pituitary microtumor 1 year later when it presented again for clinical signs of HC. One dog diagnosed with a right-sided PCC developed a second PCC 6 years later in the left adrenal gland which was then successfully removed laparoscopically. This dog developed systemic hypertension during the first surgery and hypotension in the second despite a comparable anesthetic protocol being used. Lastly, another dog developed a second AI in the contralateral adrenal gland 1.5 years after adrenalectomy, which was not removed surgically.

## 4. Discussion

Our large retrospective case series reveals high success rates of both LA and OA in dogs with functional or hormonally silent ATs. In addition, bilateral LA was shown to be a successful treatment option in dogs with HC due to bilateral adrenal neoplasia and in dogs with unilateral AT with concurrent pituitary-dependent HC. In line with previous data, recurrence of adrenal-dependent endocrinopathies occurred in a minority of dogs. In dogs with larger ATs recurrence was more common, and both DFP and survival time decreased; a correlation that has not been described previously.

Laparoscopic adrenalectomy through a flank approach in sternal recumbency was in our case series associated with a low (6.6%) conversion rate to OA and very low complication rate. The low conversion rate may be partly related to the improved surgical access to the adrenal glands because of the relatively dorsally positioned flank portals and the sternal position of the patient with hanging abdomen, moving the other abdominal organs out of the surgical field. Furthermore, this approach facilitated successful bilateral LA without repositioning or re-draping the patient.

Contrary to previously reported data (10, 16), the present study revealed no significant difference in surgical duration between LA and OA at our institution. Surgical duration is largely dependent on technical skills and experience with the technique; it is beneficial that this complex laparoscopic procedure did not result in longer surgical duration. A selection bias was demonstrated by the significantly larger AT size in OA and dogs with vascular invasion, which might be more time-consuming to remove. It was however possible to successfully remove ATs laparoscopically in comparable sized dogs as in the OA group, with the smallest dog treated laparoscopically weighing only 6.5 kg. Across both techniques, duration of right-sided adrenalectomy was significantly longer than for left-sided adrenalectomy, which can be explained by the more complex anatomy of the right adrenal gland including its more hidden location between liver and kidney and its adherence to the vena cava (9, 11). The proportion of right-sided LA in our study (49% of unilateral procedures) is relatively high compared to previous reports (29–39% right-sided LA) which might have prolonged mean surgical duration resulting in duration comparable to OA. While the actual difference in hospitalization days was small, it was significantly shorter after LA in our cohort of dogs.

This study revealed no difference in occurrence of either systemic hyper- or hypotension during LA and OA, which contrasts with the study by Taylor et al. (10). In that study, a difference was found with respect to intraoperative hypotension, occurring in 14.3 and 61.5% of dogs during LA and OA, respectively, while in the current study hypotension occurred in respectively 4.9 and 14% of dogs. This might be related to the similar surgical duration in both groups in our study, implying a comparable duration of exposure to inhalant anesthetic agents with potential blood pressure-lowering effects (32, 33). In general, CO_2_ insufflation during laparoscopic abdominal surgery has been associated with elevated arterial blood pressure (34, 35). However, with intra-abdominal CO_2_ pressure set at 8 mm Hg, no differences were found in persistent systemic hypertension or borderline hypertension during LA or OA in our study. Anesthetic complications including severe systemic hypertension have been reported specifically during removal of PCC (36). In our study, systemic hypertension and borderline hypertension were documented in only 1/8 and 2/8 dogs with PCC, respectively. In our hospital, all dogs with confirmed or highly suspected PCC are treated with phenoxybenzamine preoperatively. Based on results of a recent study one might debate whether pretreatment with this drug is beneficial, as hypertensive episodes occurred in 46/53 dogs with PCC, of which 37 were treated with an alpha-blocker before surgery (37). Numbers from that study might however be influenced by the utilized definition of systemic hypertension, as it was defined as a systolic blood pressure >160 mm Hg without specification of duration of elevation and/or methods of measurement. Blood pressure during surgery can be affected by intrinsic and extrinsic factors including effects of surgical manipulation and anesthetic drugs on venous return, cardiac output and systemic vascular resistance, depending also on anesthetic depth and intra-operative nociception. Unfortunately, no consensus exists for cut-off values that define systemic hypertension during anesthesia in dogs. It has been suggested that acute hypertension might be concerning when MAP exceeds 140 mm Hg or SAP exceeds 180 mm Hg, or in chronic hypertension when MAP exceeds 120 and/or SAP exceeds 160 mm Hg (38). Expert opinion holds that these values are just suggestions, and that decisions about treatment should be based on reliability and repeatability of the measurement, the stage of the operative procedure, the persistent nature and magnitude of blood pressure derangements, and the potential adverse consequences of the proposed treatment (38). Individual blood pressure courses documented in the current study, including short bouts of hypertension, initial hypertension with hypotension thereafter, and the occurrence of hypertension in the first, but hypotension in the second surgery in the same patient, underline the importance of close blood pressure monitoring and management by a well-trained anesthetist, in every dog during adrenalectomy.

Recurrence of adrenal-dependent endocrine disease was more common after removal of larger tumors. Possible explanations of this correlation are a more complex procedure and possible incomplete removal or tumor spillage, but also more advanced tumor development resulting in metastases which were not discovered during preoperative screening. No significant effect on DFP or recurrence was demonstrated of other perioperative parameters including surgical approach, vascular tumor invasion, lesion side and perioperative capsular penetration. The dog with recurrence and dexamethasone-suppressible HC present at diagnosis underlines the importance of including pituitary imaging and plasma endogenous ACTH measurement to avoid erroneous diagnosis of pituitary-dependent HC in dogs with bilateral adrenomegaly.

Invasion into vasculature or other tissues is considered consistent with malignancy. However, in the present study population vascular invasion was not more common in tumors with a morphological histopathologic diagnosis of carcinoma vs. adenoma. In addition, tumor size >2 cm is reported as a specific characteristic for ACC, however in this study the maximum tumor dimension exceeded 2 cm in 14/18 dogs diagnosed with ACA on histopathology. In humans, surgical removal of an ACA resulted in a better prognosis than after surgical removal of a ACC (39). In contrast, morphological histopathological parameters in dogs have not been linked to survival after removal of functional ATs (5, 6, 40). In the present study, all dogs with recurrence had a histopathologic diagnosis of ACC; however, in 23 other dogs diagnosed with carcinoma, recurrence was not reported. In this context, it is important to note that with histopathology, definitive differentiation between ACC and ACA is difficult in this species and this may have influenced the discrepancies in correlations with vascular invasion and tumor size. The same discrepancy might have been present in the dog with concurrent adrenal- and pituitary-dependent HC, in which histopathology of the suspected hyperplastic adrenal was consistent with ACC. Parameters reported to differentiate ACC from ACA in dogs are somewhat controversial and include peripheral fibrosis, capsular invasion, trabecular growth pattern, hemorrhage, necrosis, single-cell necrosis, hematopoiesis, fibrin thrombi, cytoplasmic vacuolation and increased proliferation index assessed by immunohistochemistry for the Ki67 antigen (2, 11, 12, 30). More recently, the Utrecht score was shown to be of use in distinguishing dogs with cortisol-secreting ACTs with shorter survival after adrenalectomy (29). It seems valid to include such markers of malignancy in the assessment of ATs after surgical removal, indicating the need for further studies to investigate the exact correlation with recurrence in larger groups of dogs.

In addition to recurrence of the adrenal-dependent endocrinopathy, naturally occurring food- and pituitary-dependent HC was discovered in specific cases in this study. It is therefore important to follow the diagnostic pathway as for the primary disease, to avoid incorrect diagnosis of metastatic disease rather than these alternative pathophysiologic scenarios. Two dogs developed a second PCC and AI in the contralateral adrenal gland, respectively. These are interpreted as new tumor occurrences; however, the theoretical possibility of metastatic disease cannot be excluded. As AIs can be an incidental finding during abdominal imaging for other indications than endocrine disease, repeated postoperative imaging is needed to adequately diagnose recurrence in these dogs; individual cases of recurrence might therefore have been missed in dogs without clinical signs.

The current study provides insights in the recurrence of endocrine disease in both ACT and PCC. Pheochromocytomas are reported to be more prone to invasion of the vena cava and less amenable to laparoscopic removal (10, 41). However, in the current study 5/8 PCC were successfully removed using a laparoscopic approach. In addition, no metastatic disease was diagnosed after adrenalectomy in any of the dogs with PCC. On the contrary, ACTs are described as less malignant than PCC, with distant metastasis reported to occur in only 5–14% of cases (4, 5, 16, 40), while in the current study recurrence was demonstrated in 22% discharged dogs with cortical neoplasia.

In the present study, owners of several dogs diagnosed with AI reported polydipsia at presentation. Polydipsia might be primary or secondary to unknown mechanisms induced by AI leading to polyuria. In a number of dogs, polydipsia resolved after adrenalectomy, however due to the retrospective nature of this study, it is not known whether this applied for all dogs with AI. In our study, dogs were diagnosed with AI if they failed to meet criteria as described for PCC, HC or hyperaldosteronism. This does however not imply these ATs were true silent, i.e. not hormone-secreting, tumors. The two dogs with suppressed endogenous ACTH and smaller-sized contralateral adrenal glands might have been examples secreting corticoid-precursors other than cortisol, which were not measured in the laboratory assays available. More studies are therefore needed to investigate the exact origin and endocrine effects of AI in dogs. At our institution, all dogs diagnosed with AI are treated with glucocorticoids perioperatively, while these dogs do not show overt cortisol excess during preoperative work-up as defined for HC. With this strategy any risk of corticoid-deficiency is avoided which might be relevant in cases of corticoid-precursor secretion, however, it might be unnecessary in other dogs. Postoperative ACTH-stimulation test might be useful to differentiate between these scenarios but requires further study.

Long-term survival of dogs in the current study did not differ between dogs after LA or OA, which is in line with a previous publication (10). Most studies available did not compare long-term follow-up between LA and OA. For both adrenalectomy techniques combined, 1- and 2-year survival rates of 77–83% and 60–76% are reported (10, 15, 17). Even higher percentages of 95% and 89% after LA and 92% and 88% after OA were demonstrated in the current study, supporting the conclusion of a high overall success rate of adrenalectomy in dogs.

The main limitation of this study was its retrospective nature with associated discrepancies in preoperative diagnostic work-up. In the 14 years covered in this study, availability of endocrine function tests and hormone analyses varied, leading to missing parameters in a small number of dogs. In addition, due to owner preferences, diagnostic imaging was not performed in all dogs diagnosed with recurrence based on clinical signs and endocrine function tests.

In conclusion, LA can be performed successfully in dogs of various sizes if appropriate case selection is considered and is not associated with decreased perioperative and long-term survival or recurrence. Based on low perioperative complication rate, short hospitalization time and comparable long-term outcome compared to dogs treated with OA, the less invasive laparoscopic approach is considered the preferred technique whenever possible. Higher recurrence risk in larger ATs should be considered for both techniques.

## Data availability statement

The raw data supporting the conclusions of this article will be made available by the authors, without undue reservation.

## Ethics statement

Ethical review and approval was not required for the animal study because all data reported in this manuscript was based on client-owned dogs that were treated in our hospital for their adrenal tumor(s); no additional procedures or diagnostic tests were performed. Written informed consent was obtained from the owners for the participation of their animals in this study. Written informed consent was obtained from the individual(s) for the publication of any potentially identifiable images or data included in this article.

## Author contributions

KB, SG, HK, and SN contributed to conception and design of the study. KB, SG, and SN organized the database. ET and KB performed the statistical analysis. KB wrote the first draft of the manuscript. JG, GG, ET, and SN wrote sections of the manuscript. All authors contributed to manuscript revision, read, and approved the submitted version.
